# Coordination of Mg^2+^ with Chitosan for Enhanced Triboelectric Performance

**DOI:** 10.3390/polym17081001

**Published:** 2025-04-08

**Authors:** Jingjia He, Lili Wang, Kaiyuan Zheng, Shoukang Hu, Xueke Zhang, Ziyu Mu

**Affiliations:** College of Chemistry, Chemical Engineering and Resource Utilization, Northeast Forestry University, Harbin 150040, China; 19917627698@163.com (J.H.); z202109130101@163.com (K.Z.); hsk17739621640@163.com (S.H.); 19811713786@163.com (X.Z.); mzy121382023@163.com (Z.M.)

**Keywords:** Triboelectric Nanogenerator (TENG), chitosan, magnesium ion (Mg^2+^), coordination, charge transfer

## Abstract

In this work, Mg^2+^ modified chitosan (Mg^2+^/CS) is proposed and successfully designed. By investigating the effects of the Mg^2+^ and CS interaction on hydrogen bonding, dipoles, charge density, surface potential, and roughness, the coordination between Mg^2+^ and CS is verified and the mechanism of coordination improving tribological properties is elucidated. The Mg^2+^/CS coordination structure enhances intermolecular interactions, promoting the formation of new hydrogen bonds and increasing the dipoles. Compared to CS, the relative dielectric constant of Mg^2+^/CS increased by 76%, the surface potential increased by 70 mV, and the root mean square roughness increased by 39.4 nm. The open-circuit voltage, short-circuit current, and charge density of the triboelectric nanogenerator (TENG) fabricated from Mg^2+^/CS were increased by 100%, 94%, and 75%, respectively, compared to the CS-TENG fabricated from pure CS. The coordination of Mg^2+^ increased the charge density of the Mg^2+^/CS-TENG, significantly enhancing its charge transfer capability. The Mg^2+^/CS-TENG successfully provided power for photodetectors and LEDs. Mg^2+^/CS exhibited excellent flexibility and skin adhesion, and the Mg^2+^/CS-TENG successfully converted the mechanical energy generated by human joint motion into electrical signals. The coordination structure of Mg^2+^ with CS enhances the triboelectric performance of Mg^2+^/CS-TENG, providing new light for the research of chitosan-based TENGs.

## 1. Introduction

Triboelectric Nanogenerator (TENG) is an emerging technology that converts mechanical energy into electrical energy through the coupling effects of the triboelectric effect and electrostatic induction [1,2,3]. The continuous advancement of the TENG technology is expanding its application fields. Especially in self-powering and flexible electronic skins [4], TENG provides a green and environmentally friendly energy harvesting solution and meets the requirement for highly integrated and miniaturized devices through diversified designs. As the demand for green energy and resource efficiency increases in today’s society, natural biomaterials have gradually gained favor among researchers due to their advantages, such as wide availability, renewability, and eco-friendliness.

Chitosan (CS) is one of the most abundant natural biomaterials found in the exoskeletons of crustaceans [5]. Due to its advantages, such as natural biodegradability, good biocompatibility, and non-toxicity, it has been widely studied in many biomedical applications [6,7,8,9]. The abundant primary hydroxyl (−OH) and secondary amine (−NH_2_) groups on CS tend to release electrons and can act as the positive electrode material in TENGs [10]. However, due to the low surface charge density of CS, its triboelectric output performance is severely affected. Hu et al. composited CS with silver nanoparticles, and the resulting CS/Ag had an open−circuit voltage (V_OC_) of 74 V and an open−circuit current (I_SC_) of 4.6 μA [11]. Pongampai et al. added lead-free piezoelectric BaTiO_3_ nanorods into CS, achieving a maximum V_OC_ of 111.4 V, an I_SC_ of 21.6 μA/cm^2^, and an output power density of 756 μW/cm^2^ [12]. Hu et al. mixed TiN nanoparticles with CS to prepare TiN/CS films, which exhibited a V_OC_ of 67 V, an I_SC_ of 4.2 μA, and a charge transfer of 18.5 nC [13]. The reports above mainly focus on improving the output performance by physically compositing Ag or inorganic nanofillers with CS.

Fortunately, the impact of changes in the internal bonding interactions of CS on its triboelectric performance has also received attention. Charoonsuk et al. found that the bonding interactions between Ca^2+^ and CS/glycerol generate more electrons and unoccupied orbitals, which enhances the charge density and charge transfer during the triboelectric process [14]. Wang et al. reported that CS with Ag nanowires crosslinked Ag^+^ and Cu^2+^ separately, investigating the impact of different charge transfer mechanisms on the triboelectric performance [15]. Chaturvedi et al. reported a composite film of CS and dry-leaf-waste-activated carbon as a triboelectric material, where the coordination interaction between Na^+^ and the -OH of CS affected the charge generation and transfer [16]. Mg^2+^ exhibits good ionic conductivity [17] and electrophilicity [18], and is widely used in electrochemical catalysis [19], fuel cells [20], and supercapacitors [21]. Moreover, Mg^2+^ is cost-effective and has environmental advantages over other heavy metal ions. However, the effect of the bonding interactions between Mg^2+^ and the abundant −NH₂ and −OH groups of CS on the charge density and electron transfer in Mg^2+^/CS has not been reported. Based on the above discussion, coordination between Mg^2+^ and CS is assumed. The coordination interaction is hypothesized to modulate electron cloud density and elevating the relative constant, which enhance the triboelectric performance of CS.

In this work, Mg^2+^ modified CS was designed. MgO was used as the magnesium source and reacted in situ with the solvent acetic acid, which dissolves CS, to prepare Mg^2+^/CS without introducing new anions. To validate the hypothesis proposed herein, The effect of the bonding interactions between Mg^2+^ and CS on charge density and surface potential has been investigated. The experimental results show that new hydrogen bonds are formed in Mg^2+^/CS, which increases the dipole density and enhances the charge transfer capability. Compared to CS, the relative dielectric constant of Mg^2+^/CS increases from 1068 to 1885, the surface potential increases from 895 mV to 965 mV, and the root mean square roughness increases from 26.3 nm to 65.7 nm. The V_OC_, I_SC_, and charge density (σ_SC_) of the Mg^2+^/CS−TENG are 112 V, 3.7 μA, and 2.1 nC/cm^2^, respectively. Utilizing the flexible characteristics of the Mg^2+^/CS film, the Mg^2+^/CS−TENG was used to harvest electrical signals from human body movements such as bending fingers, wrist vibrations, and fist clenching, demonstrating its application in wearable electronic devices. Meanwhile, the fabricated Mg^2+^/CS−TENG successfully powered a small commercial photodetector and lit up 63 blue LEDs. These demonstrations underscore the potential of Mg^2+^/CS−TENG in self-powered applications, opening new avenues for combining sustainable energy with intelligent technology.

## 2. Materials and Methods

### 2.1. The Preparation of CS/Mg^2+^

A total of 1 g of CS (degree of deacetylation ≥ 95, viscosity 100–200 mPa·s, Rhawn, Shanghai, China) powder was dissolved in 2% acetic acid (99% reagent grade, Aladdin, Shanghai, China) and stirred at 60 °C for 4 h to obtain a uniform, light−yellow, transparent solution. A total of 0.4 g of MgO (99.9% purity, Aladdin, Shanghai, China) was dispersed in 10 mL of deionized water. The MgO dispersion was then added to the CS solution and stirred at 60 °C for 2 h. The composite film of the above mixture was prepared via micro−electronic printer (Module DB100, Shanghai Mifang Electronic Technology, Shanghai, China). The obtained sample film was labeled as Mg^2+^/CS.

### 2.2. The Fabrication of Mg^2+^/CS−TENG

**Figure 1:**
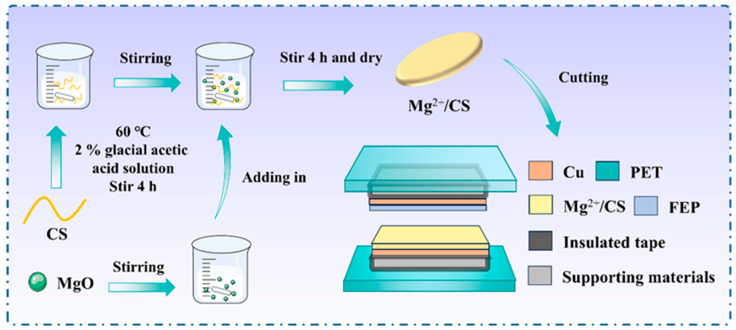
Preparation of Mg^2+^/CS and the device structure of Mg^2+^/CS−TENG.

The Mg^2+^/CS and FEP were cut into sizes of 2.5 cm × 3.5 cm, and copper foils were attached to the back of the films as the positive and negative electrodes, respectively. Two pieces of PET sheets (10 cm × 10 cm) and two pieces of sponge (5 cm × 5 cm) were cut. Double−sided adhesive tape was applied to the two opposite surfaces of the PET sheets. Springs were attached to the four corners of the sheets. The surface of the sponge was wrapped with insulating tape and fixed onto the PET sheets, and electrical wires and copper plates were attached to the top of the sponge. Finally, the positive and negative electrodes were fixed onto the sponge to obtain Mg^2+^/CS−TENG. The preparation process of the Mg^2+^/CS film and the device structure of Mg^2+^/CS−TENG are shown in Figure 1.

### 2.3. Characterization and Measurement

The crystallinity and crystal structure of the samples were characterized by X−ray diffraction (XRD, SmartLab SE, Rigaku Corporation, Tokyo, Japan). The tube voltage is 40 kV and the tube current is 40 mA. The functional groups of the samples were characterized by Fourier Transform Infrared spectrometer (FTIR, Nicolet IS10 ThermoFisher Scientific Corporation, Waltham, MA, USA). Scanning Electron Microscopy (SEM, A JSM−7500F, Hitachi, Tokyo, Japan) observed the composite films’ morphology at an accelerating voltage of 10 kV. The surface roughness of the materials was measured by Atomic Force Microscopy (AFM, MFP−3D, Asylum Research, Santa Barbara, CA, USA). The surface potential of the sample was measured by Kelvin probe force microscopy (KPFM, BRUKER Dimension Iconce, Bruker Corporation, Billerica, MA, USA). The elemental composition, valence state and chemical environment of the material were characterized by X−ray photoelectron spectroscopy (XPS, ESCALAB 250Xi, Thermo Fisher Scientific Corporation, Waltham, MA, USA). The dielectric properties at room temperature were characterized by dielectric performance tester (Agilent 4294A, Agilent Technologies, Palo Alto, CA, USA). The mechanical properties of the composite film were tested by an electronic universal testing machine (MTS E43.104, MTS System Corporation, Eden Prairie, MN, USA), and a linear motor system (LinMot E1100, LinMot GmbH, Baden−Württemberg, Germany) was used to fix the frequency. The open−circuit voltage, short−circuit current, and charge density of the TENG were measured by an electrometer (Keithley 6514, Keithley Instruments, Cleveland, OH, USA). A mixed−domain oscilloscope (Tektronix 2012C, Tektronix, Beaverton, OR, USA, impedance = 1 MΩ) was used to monitor the voltage output signal.

## 3. Results and Discussion

**Figure 2:**
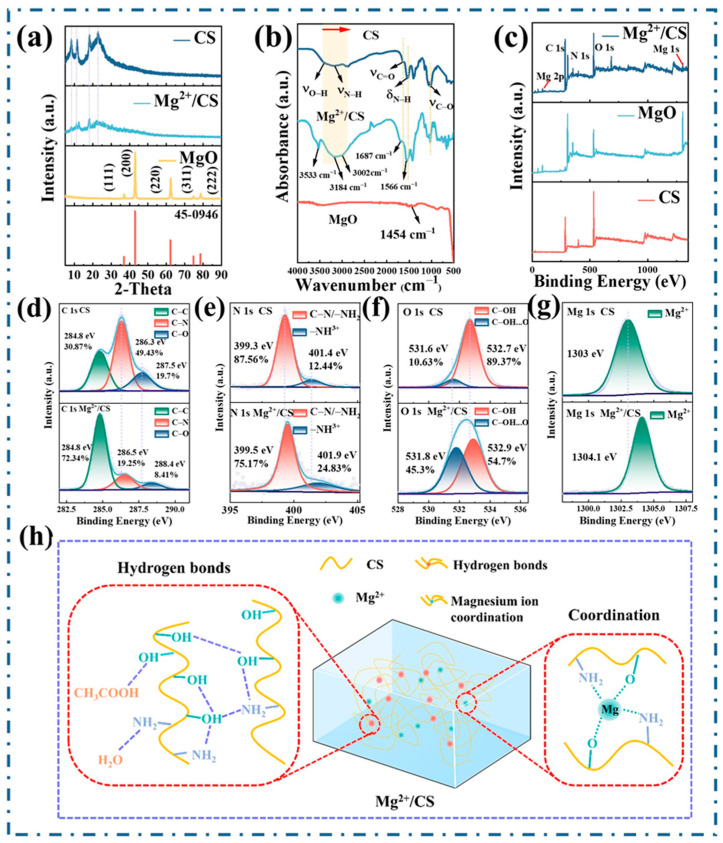
XRD patterns, FTIR spectra, XPS spectra of CS and Mg^2+^/CS: (**a**) XRD patterns of CS, Mg^2+^/CS, and MgO, (**b**) FTIR of CS, Mg^2+^/CS, and MgO, (**c**) XPS survey scan spectra of CS, MgO, and Mg^2+^/CS, (**d**) C 1s binding energy spectra of CS and Mg^2+^/CS, (**e**) N 1s binding energy spectra of CS and Mg^2+^/CS, (**f**) O 1s binding energy spectra of CS and Mg^2+^/CS, (**g**) Mg 1s binding energy spectra of MgO and Mg^2+^/CS, (**h**) internal bonding of Mg^2+^/CS.

Figure 2a presents the XRD patterns of CS, MgO, and Mg^2+^/CS. The characteristic diffraction peaks of CS were observed at 8.3°, 11.5°, 18.1°, and 22.9° [22]. For MgO, diffraction peaks corresponding to the (111), (200), (220), (311), and (222) planes are observed at 36.9°, 42.9°, 62.3°, 74.7°, and 78.6°, respectively, consistent with the cubic phase of MgO as indicated by the standard JCPDS file (45−0946) [23]. Compared to pure CS, the intensity of peaks at 11.5° and 22.9° in Mg^2+^/CS is reduced, suggesting a decrease in the crystallinity of CS [24]. Notably, no characteristic peaks of MgO are present in the XRD pattern of Mg^2+^/CS, confirming that MgO is absent, as anticipated. This result indicates that MgO has reacted with the acetic acid used to dissolve CS, leading to the formation of Mg^2+^.

To verify the formation of the coordination structure, the FTIR spectra of CS, MgO, and Mg^2+^/CS are presented in Figure 2b. The absorption bands of CS at 3359 cm^−1^ and 3208 cm^−1^ are attributed to the stretching vibrations of the O−H and N−H bonds, respectively [25]. The bands at 2925 cm^−1^ and 2879 cm^−1^ correspond to the asymmetric and symmetric stretching vibrations of the C−H bonds. The 1636 cm^−1^ and 1535 cm^−1^ bands are assigned to the C=O stretch and the N−H bend, respectively. The absorption band at 1020 cm^−1^ is due to the stretching vibration of the C−O bond. MgO exhibits an O−H bending absorption peak at 1454 cm^−1^ [26]. Furthermore, the characteristic peak of MgO is absent in Mg^2+^/CS, providing evidence for the absence of MgO. The coordination between CS and Mg^2+^ induces significant changes in the infrared spectrum of Mg^2+^/CS. Compared with the O−H band of CS, the blue−shifted peak at 3533 cm^−1^ of Mg^2+^/CS can be ascribed to the coordination−induced reduction in electron density of the O−H bond and the formation of weak hydrogen bonds [27,28]. Conversely, the O−H peak at 3184 cm^−1^ and the N−H peak at 3002 cm^−1^ both exhibit a notable redshift compared to CS, indicating the formation of new hydrogen bonds [29]. Within the 1700–1500 cm^−1^ range in Mg^2+^/CS, compared to pure CS, both the C=O stretch and the N−H bend bonds exhibit shifts. The increased polarity of the C=O bond, resulting from the electrostatic attraction of the carbonyl oxygen’s lone pair electrons by the positively charged Mg^2+^, enhances its vibrational frequency, leading to a blue shift [30]. The shift in the N−H bend is indicative of its participation in metal coordination, as the functional group absorption peak may shift either upfield or downfield upon complexation; this observation confirms the involvement of the amino group in metal ion coordination [31].

To further confirm the coordination of CS/Mg^2+^, XPS spectra of Mg^2+^/CS, CS, and MgO are shown in Figure 2c–g. The full XPS spectrum of Mg^2+^/CS reveals the presence of C, O, N, and Mg elements (Figure 2c). The C 1s binding energy for C−C, C−N, and C−O in Mg^2+^/CS are 284.8 eV, 286.5 eV, and 288.4 eV, respectively (Figure 2d). Compared to CS, the binding energy for C−N and C−O are increased by 0.2 eV and 0.9 eV, respectively, from the values of 286.3 eV and 287.5 eV observed in CS [32]. The N 1s binding energy for −NH_2_/C−N and −NH_3_^+^ in Mg^2+^/CS are 399.5 eV and 401.9 eV, respectively (Figure 2e), showing increases of 0.2 eV and 0.5 eV compared to the binding energy for −NH_2_/C−N (399.3 eV) and −NH_3_^+^ (401.4 eV) in CS. The O 1s binding energy for C−OH and C−OH...O in Mg^2+^/CS are 531.8 eV and 532.9 eV, respectively (Figure 2f), which are both 0.2 eV higher than those observed in CS (531.6 eV and 532.7 eV, respectively). During the coordination of Mg^2+^ with the −NH_2_ and −OH groups of CS, the partial transfer of lone−pair electrons from the ligands to the vacant orbitals of Mg^2+^ reduces the local electron density of C, N, O atoms, resulting in an increase in binding energies of C, N, O [33].

For MgO, the Mg 1s binding energy is 1303.0 eV (Figure 2g), while in Mg^2+^/CS, the Mg 1s the binding energy shifts to 1304.1 eV, a 1.1 eV increase compared to MgO. The electrons are transferred from CS to Mg^2+^, so the electron density around Mg^2+^ increases. However, the reasons for the change in binding energy of Mg is not only from a simple increase in electron density, but also controlled by the charge redistribution and polarization effects induced by coordination bonding. The formation of the Mg^2+^− ligand coordination bonds triggers polarization within the CS molecular chains due to the Mg^2+^. This polarization redistributes local electron clouds, thereby weakening the electron shielding effect. The amplified local electric field intensity around Mg^2+^, caused by this polarization, ultimately leads to a significant increase in the Mg 1s binding energy. In summary, the polarization effects and charge redistribution driven by coordination bonding are the core mechanisms responsible for the binding energy shifts, while the reduced electron density of the ligands represents a localized manifestation of this process [34,35].

The content of C−OH...O in CS is 10.63%, while it increases significantly to 45.30% in Mg^2+^/CS (Figure 2f), highlighting a marked enhancement of hydrogen bonding in the coordination compound. FTIR and XPS results confirm the existence of coordination interactions between CS and Mg^2+^, accompanied by increased hydrogen bonding. The hydrogen bonds in Mg^2+^/CS may include intramolecular hydrogen bonds between the −OH and −NH_2_ groups in CS, intermolecular hydrogen bonds, and hydrogen bonds between CS and the solvent molecules (acetic acid, water). The coordination structure increases electron density, strengthens intermolecular interactions, and facilitates the formation of new hydrogen bonds [36,37]. Mg^2+^ induces electron cloud redistribution within CS molecules, creating significant dipole moments by separating positive and negative charge centers, thereby enhancing the charge transfer capacity between functional groups and generating localized high−polarity regions. This polarization effect not only strengthens electrostatic interactions within the polymer matrix but also disrupts the original hydrogen bond equilibrium. The newly formed high−polarity zones promote hydrogen bond reformation through enhanced electrostatic attraction between neighboring functional groups [34,38]. The coordination bonds formed between CS and Mg^2+^, interwoven with hydrogen bonds, establish a charge transport network. The bonding schematic is illustrated in Figure 2h.

**Figure 3:**
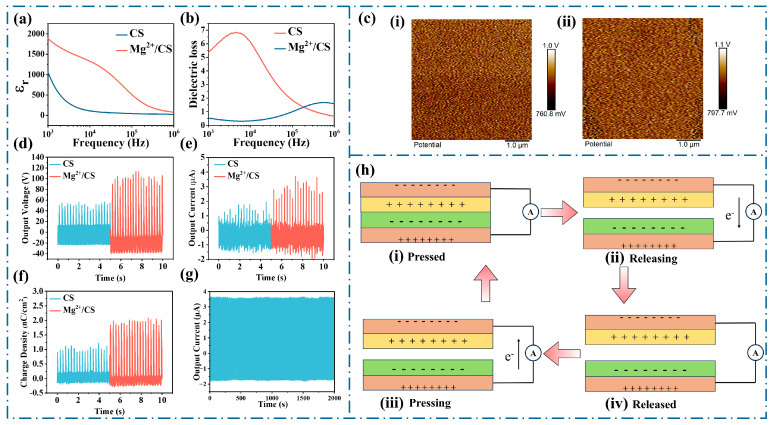
(**a**) Relative dielectric constant of CS and Mg^2+^/CS, (**b**) dielectric loss of CS and Mg^2+^/CS, (**c**) KPFM image at the 1 μm scale: (**i**) CS (**ii**) Mg^2+^/CS, (**d**–**f**) V_OC_, I_SC_, and σ_SC_ of CS and Mg^2+^/CS, (**g**) Stability test of Mg^2+^/CS, (**h**) stepwise working mechanism of the Mg^2+^/CS−TENG, in the figure, orange represents copper, yellow represents CS, and green represents FEP.

To verify the enhancement of polarity in Mg^2+^/CS, the relative dielectric constant and dielectric loss curves of CS and Mg^2+^/CS are presented in Figure 3a,b, respectively. At low frequencies, the relative dielectric constant of CS is 1068, while that of Mg^2+^/CS increases to 1885, representing a 76% increase. This enhancement in the relative dielectric constant can be attributed to the higher number of hydrogen bonds in Mg^2+^/CS, which strengthens the dipolar interactions [39,40]. The relative dielectric constant also exhibits a frequency dependence, with a decrease observed at higher frequencies. This is due to the dipoles’ inability to follow the electric field’s rapid variations, leading to a reduction in relative dielectric constant at elevated frequencies. Concurrently, the dielectric loss increases slightly, reflecting the enhanced dipole polarization [41]. The charge density of triboelectric materials is related to their relative dielectric constant. The relationship between the maximum charge density of triboelectric materials and their relative dielectric constant is expressed in Equation (1) [42].

(1) $$\sigma^{\prime }=\frac{\sigma_{0}d_{\mathrm{gap}}}{d_{\mathrm{gap}}+d_{CS}/\varepsilon_{CS}}$$

In this equation, *ε_CS_*, *d_CS_*, *d*_gap_, and *σ*_0_ correspond to the relative dielectric constant of the material, the thickness of the film, the distance between the triboelectric electrodes, and the surface charge density of the triboelectric material at equilibrium, respectively. The equation shows that the relative dielectric constant is directly proportional to the surface charge density. Therefore, an increase in the relative dielectric constant facilitates a higher surface charge density.

To confirm that the surface charge of Mg^2+^/CS increases compared to CS, KPFM images are shown in Figure 3c,d. The surface potential of the CS film is 895 mV, while the coordination of Mg^2+^ raises the surface potential of Mg^2+^/CS to 965 mV. The trends observed in the relative dielectric constant and surface potential are consistent, indicating that the increase in dipoles contributes to the enhancement of the surface charge. It can be inferred that the rise in surface potential results from the increased charge density.

Triboelectric generation primarily relies on the coupling of contact electrification and electrostatic induction effects. Its operating mechanism is described as a “press−release” cycle consisting of four continuous stages. Figure 3h illustrates the working principle of the Mg^2+^/CS−TENG. The Mg^2+^/CS film is the positive triboelectric material. Upon contact between the positive and negative electrode materials (Figure 3h(i)), surface charges are generated through electrification, accumulating negative charges on the FEP deposition layer and positive charges on the Mg^2+^/CS surface. As the contact pair begins to separate (Figure 3h(ii)), electrostatic induction induces the transfer of opposite charges to each electrode, causing the current to flow through the external circuit from the positive to the negative electrode. Once the films are fully separated, charge equilibrium is reached on both sides of the electrodes (Figure 3h(iii)). As the films begin to approach each other again, the direction of the current flow is reversed, moving from the negative to the positive electrode (Figure 3h(iv)). Thus, the contact and separation cycle between the paired films generates alternating output performance [43].

The V_OC_, I_SC_, and σ_SC_ curves of CS and Mg^2+^/CS are presented in Figure 3d–f, respectively. For CS, the V_OC_ is 55.9 V, I_SC_ is 1.9 μA, and σ_SC_ is 1.2 nC/cm^2^. In contrast, for Mg^2+^/CS, V_OC_ increases to 113.1 V, I_SC_ to 3.7 μA, and σ_SC_ to 2.1 nC/cm^2^. Compared to CS, the V_OC_, I_SC_, and σ_SC_ of Mg^2+^/CS are enhanced by 100%, 94%, and 75%, respectively. The increase in σ_SC_ is consistent with the observed improvements in the relative dielectric constant and surface potential, which can be attributed to the enhanced dipole interactions that facilitate the charge transfer during the triboelectric charging process [44]. As shown in Figure 3g, Mg^2+^/CS demonstrates stable voltage signals throughout a 2000 s cycling test, further confirming its reliability.

**Figure 4:**
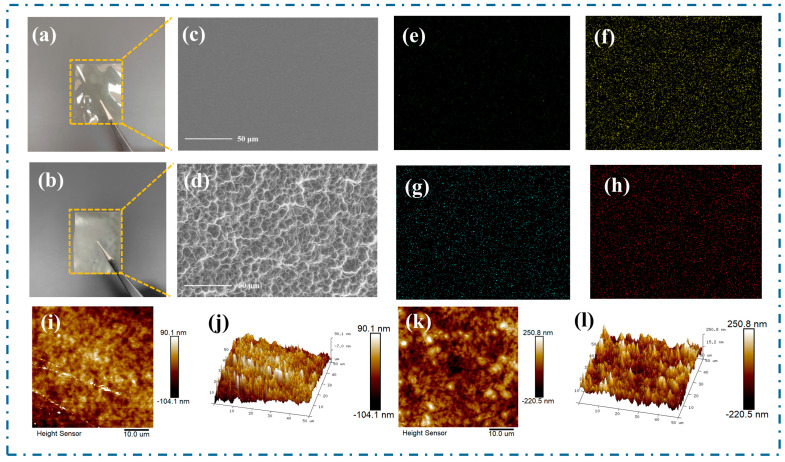
Photographs and SEM, EDS, and AFM images: (**a**) photograph of CS, (**b**) photograph of Mg^2+^/CS, (**c**) SEM image of CS at 50 μm scale, (**d**) SEM image of Mg^2+^/CS at 50 μm scale, (**e**–**h**) EDS images of N, O, Mg, and C in the Mg^2+^/CS, (**i**,**j**) AFM images of CS at 10 μm scale, (**k**,**l**) AFM images of Mg^2+^/CS at 10 μm scale.

Figure 4c,d present the SEM images of the CS and Mg^2+^/CS films at a 50 μm scale, respectively. The CS film exhibits a smooth and uniform morphology. In contrast, the surface of Mg^2+^/CS displays a rough, polygonal, and irregular structure, which is attributed to the formation of the coordination compound. Figure 4e–h further shows the elemental mapping images of N, O, Mg, and C in Mg^2+^/CS. As indicated in Figure 4g, the Mg element was well dispersed in the acquired region, confirming the successful incorporation of Mg^2+^ into CS.

The change in the surface roughness of materials can influence triboelectric properties [45]. To assess the impact of Mg^2+^ addition on the film’s surface roughness, AFM images of CS and Mg^2+^/CS are shown in Figure 4i–l. The root mean square (RMS) roughness of Mg^2+^/CS is 65.7 nm, significantly higher than the 26.3 nm of the CS film. The increased surface roughness of Mg^2+^/CS enhances the contact area between the Mg^2+^/CS and the negatively charged electrode material (FEP), thereby contributing to the improved triboelectric performance of the Mg^2+^/CS−TENG.

**Figure 5:**
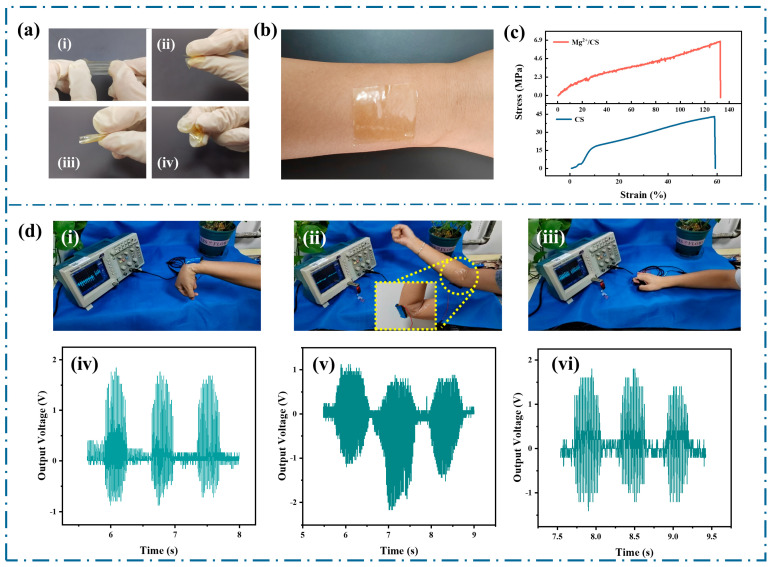
Mechanical properties and applications of Mg^2+^/CS: (**a**) flexibility demonstrations of Mg^2+^/CS: (**i**) tension, (**ii**) folding, (**iii**) curling, (**iv**) kneading, (**b**) skin adhesion demonstration of Mg^2+^/CS, (**c**) stress–strain curves of CS and Mg^2+^/CS, (**d**) application of Mg^2+^/CS−TENG in wearable electronic devices: (**i**,**iv**) wrist joint, (**ii**,**v**) elbow joint, (**iii**,**vi**) finger joint.

Figure 5a presents digital photos of the Mg^2+^/CS film undergoing various deformations, including stretching, folding, curling, and kneading. Figure 5b highlights the excellent transparency and skin adhesion of the Mg^2+^/CS film. The stress–strain curves of CS and Mg^2+^/CS are shown in Figure 5c. CS reaches a strain of 59.3% at a stress of 43.2 MPa. In contrast, the Mg^2+^/CS film exhibits significantly enhanced stretchability, achieving a strain of 132.8% at 6.76 MPa, which is 2.24 times greater than that of CS.

Figure 5d illustrates the electrical output signal generated by the Mg^2+^/CS−TENG when fixed to the tester’s joints. During wrist, elbow, and finger flexion movements, the Mg^2+^/CS−TENG produces corresponding voltage outputs of 2 V, 1 V, and 1.5 V.

**Figure 6:**
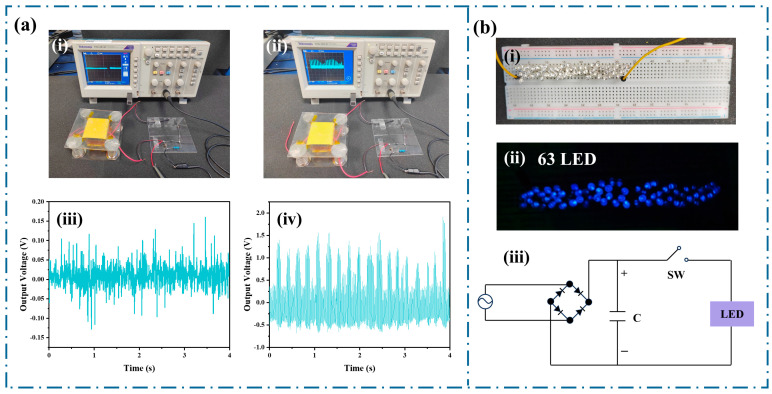
Application of Mg^2+^/CS−TENG in self−powered systems, (**a**) Mg^2+^/CS−TENG driving a small photodetector: (**i**) photograph of the device in a static state, (**ii**) voltage change on the oscilloscope after pressing Mg^2+^/CS−TENG for power supply, (**iii**) voltage across the photodetector in a static state, (**iv**) voltage across the photodetector after pressing Mg^2+^/CS−TENG to drive it, (**b**) Mg^2+^/CS−TENG driving the LED: (**i**) LED not lit, (**ii**) LED light up after pressing Mg^2+^/CS−TENG, lighting 63 LEDs, (**iii**) circuit diagram of Mg^2+^/CS−TENG driving the LED.

Figure 6 demonstrates the applications of Mg^2+^/CS−TENG in powering various electronic devices. Figure 6a shows the Mg^2+^/CS−TENG driving a commercial small photodetector. In the stationary state (Figure 6a(i,iii)), the electrostatic potential is approximately 0.1 V. When the photodetector is powered by being pressed (Figure 6a(ii)), the Mg^2+^/CS−TENG generates a voltage of around 1.5 V (Figure 6a(iv)). Figure 6b shows the Mg^2+^/CS−TENG successfully powered 63 blue LEDs.

Table 1 presents the comparative analyses of the V_OC_ and I_SC_ values of TENGs reported in various studies. Except for the V_OC_ value of chitosan−diatom (150 V) [46] and the I_SC_ value of CS/AgNWs (4.1 mA) [42], which are higher than that of the Mg^2+^/CS in this study, the other reported values are lower than the triboelectric output of the Mg^2+^/CS−TENG [10,47,48].

## 4. Conclusions

In summary, this work fabricated Mg^2+^/CS films and investigated the influence of molecular internal bonding on triboelectric performance by modifying the chitosan molecular structure. The experimental results confirm that the hypothesis is reasonable; the coordination structure between Mg^2+^ and CS enhances intermolecular interactions by altering electron density, thereby promoting the formation of hydrogen bonds, increasing dipoles, improving charge density, and enhancing charge transfer capabilities. Compared to the CS−TENG, the V_OC_, I_SC_, and σ_SC_ of the Mg^2+^/CS−TENG were enhanced by 100%, 94%, and 75%, respectively. Moreover, successful applications have been achieved in self-powered systems and activity signal monitoring. However, the material structure changes and energy conversion mechanism changes that Mg^2+^/CS may cause in extreme temperature environments have not been systematically explored, such as extremely hot or cold environments. This work demonstrates that metal-ion coordination represents a simple and effective strategy to enhance the triboelectric performance of CS−based materials. By introducing metal-ion coordination to other biopolymers rich in active functional groups, the electron distribution and dipole arrangement of polymer chains can be precisely regulated, enhancing interfacial polarization and charge capture ability. This method provides a new universal strategy for developing high-performance, entirely biomass-based triboelectric electric nanogenerators.

## Figures and Tables

**Table 1:** Comparison with other triboelectric material research work.

Main Material	Friction Material	S (cm)	Triboelectric Property				Test Conditions
			VOC		ISC		
chitosan-diatom [46]	FEP	3 × 4	150 V	↓	1.0 μA	↑	8 N
CS/AgNWs [42]	PVDF	2 × 2	47.9 V	↑	4.1 mA	↓	3 Hz
Chitosan/chondroitin Sulfate/ZnO [10]	FEP	2.5 × 4	105 V	↑	3.3 μA	↑	30 N, 1 Hz
CS/PVA [47]	Rice paper	2.5 × 2.5	20 V	↑	200 nA	↑	5 N 1 Hz
CS/PVA [48]	PVDF	3 × 3	20.8 V	↑	878 nA	↑	5 Hz
Mg^2+^/CS (our work)	FEP	2.5 × 3.5	113.1 V		3.7 μA		10 N

The “↑” indicates better performance for this job, and the “↓ ”means the opposite.

## Data Availability

The original contributions presented in this study are included in the article. Further inquiries can be directed to the corresponding author.
